# Sex- and age-specific variations, temporal trends and metabolic determinants of serum uric acid concentrations in a large population-based Austrian cohort

**DOI:** 10.1038/s41598-020-64587-z

**Published:** 2020-05-05

**Authors:** Emanuel Zitt, Anton Fischer, Karl Lhotta, Hans Concin, Gabriele Nagel

**Affiliations:** 1 Vorarlberg Institute for Vascular Investigation and Treatment (VIVIT), Academic Teaching Hospital Feldkirch, Feldkirch, Austria; 2 Department of Internal Medicine III (Nephrology and Dialysis), Academic Teaching Hospital Feldkirch, Feldkirch, Austria; 3 Agency for Preventive and Social Medicine, Bregenz, Austria; 4 Institute for Epidemiology and Medical Biometry, Ulm University, Ulm, Germany

**Keywords:** Nephrology, Risk factors, Signs and symptoms

## Abstract

Little is known about sex- and age-specific variations and temporal trends in serum uric acid (SUA) concentrations, the prevalence of hyperuricemia and its association with metabolic risk factors in the general population. Between January 1, 1985 and June 30, 2005 146,873 participants (42% women) were recruited. Prevalence of hyperuricemia was estimated applying a common (SUA > 360 µmol/L) and sex-specific cut-off points (women > 340 µmol/L, men > 420 µmol/L). At baseline, mean age was 41.2 years in men and 51.5 years in women, mean SUA concentration was 314.8 µmol/L and 243.6 µmol/L, respectively. Applying a common cut-off point, the prevalence of hyperuricemia was 18.5% in men and 4.4% in women and by sex-specific cut-off points it was 15.1% and 13.8%, respectively. SUA levels increased by 6.7 µmol/L per decade in men, but remained constant in women until the age of 50 years with a sharp increase by approximately 22 µmol/L per decade thereafter. In men and women, hyperuricemia was associated with obesity, hypertriglyceridemia and elevated gamma-glutamyl transferase. With increasing age SUA levels and the prevalence of hyperuricemia rise in a sex-specific manner. Above the age of 65 years, the sex-specific prevalence of hyperuricemia in women outreaches that in men.

## Introduction

Uric acid (UA) is generated by the metabolic breakdown of purine nucleotides and nucleosides such as adenosine. In humans and great apes it is the final degradation product, whereas in other mammals UA is further oxidized to allantoin. Uricase, the enzyme catalyzing this process, has been silenced during human evolution by nonsense mutations^1^. Therefore, humans have three to ten times higher serum UA (SUA) levels than do other species. Urate is excreted by the kidney by free glomerular filtration with complex reabsorption and secretion in the proximal tubulus^2^.

It is hypothesized that the powerful antioxidant capacity of UA may have caused a survival advantage during human evolution^3^. On the other hand, high UA levels have been shown to be associated with hypertension, cardiovascular disease, metabolic syndrome, diabetes and chronic kidney disease, making it a valuable biomarker for these conditions^4–9^. Furthermore, UA levels and prevalence of hyperuricemia seem to have increased during recent decades and years^10–13^. Therefore, from a public health perspective knowledge and surveillance of SUA levels in the general population are valuable goals.

The aim of the present study was to describe sex- and age-specific variations and temporal trends in SUA concentrations, the prevalence of hyperuricemia and its association with metabolic risk factors in a large population-based cohort over a follow-up of 20 years.

Herein, we present SUA levels obtained during a period of over 20 years for 146.873 persons with over 530.000 individual SUA measurements collected between 1985 and 2005 in Vorarlberg, the westernmost state of Austria. We describe sex- and age-specific variations in SUA levels as well as the prevalence of hyperuricemia and the associations with other metabolic risk factors.

## Methods

### Study population

The Vorarlberg Health Monitoring & Prevention Programme (VHM&PP) is a population-based risk factor surveillance program in Vorarlberg. The VHM&PP cohort has been described in more detail elsewhere^14,15^. The Agency for Social and Preventive Medicine (aks) invited all adults in Vorarlberg (population: approx. 400.000) to participate in the health surveillance program including a standardized interview, examination and blood sampling. Enrolment was voluntary, and the costs for one examination per year were covered by the participant’s compulsory health insurance. Between January 1, 1985 and June 30, 2005 185,341 persons had at least one health examination (HE). In 146,873 participants SUA was measured at enrolment. In men, SUA was measured in all age groups. In women, however, SUA was routinely determined only in those aged ≥50 years. In total, data from 530,721 SUA measurements (223,738 in women and 306,983 in men) were available for this analysis. In median 3 (Q1, Q3; 1, 5) measurements were available.

The screening examinations were conducted in the practices of local physicians according to a standard protocol. Height, body weight, systolic blood pressure (BPsys) and diastolic blood pressure (BPdia) were determined. Information on smoking status was collected in a standardized interview. Blood glucose (BG), total cholesterol (TC), triglycerides (TG) and gamma-glutamyl transferase (GGT) were measured (since January 1, 1988) in an overnight fasting blood sample. In two central laboratories with regular internal and external quality procedures, the blood was centrifuged for 15 minutes at 4.000 rotations per minute. SUA concentrations were measured on an RXL (DADE). Calibration was checked using three daily control samples. If the average values of the control samples were not within 3% of the true value, the run was repeated. Day-by-day variation had to be within 5%^16^.

Hyperuricemia was defined according to two commonly used definitions: 1) a common cut-off point of 360 µmol/L for women and men, 2) a sex-specific cut-off point of 340 µmol/L for women and 420 µmol/L for men^11,17^.

Obesity was defined according to the WHO definition as BMI ≥ 30 kg/m². Hypertension was defined as systolic blood pressure ≥140 mmHg or diastolic blood pressure ≥90 mmHg^18^.

### Statistical analysis

The distribution of SUA concentration at baseline was described by means and standard deviation. The density of SUA was calculated with kernel density estimation.

A generalized linear model was calculated for SUA and BMI as response variable and age as explanatory variable. Data from different subjects are assumed to be statistically independent, and data within subjects are assumed to be equally correlated. To account for the bias of time-dependent explanatory variable, least squares means were calculated for age.

To compare the distribution of SUA concentrations and BMI at baseline for different decades a generalized linear model was calculated for SUA as response variable and time period as explanatory variable. Data from different time periods are assumed to be statistically independent, as well as data within time periods. To account for the bias of time-dependent explanatory variable, the model was adjusted for age. Participants were classified in five time-period groups of five years each (Year 1980–1984, …, Year 2000–2004) according to the year of first health examination.

The point prevalence of hyperuricemia was calculated with the following equation:

$${\rm{Point}}\,{\rm{prevalence}}\,({\rm{ratio}})=\frac{{\rm{Number}}\,{\rm{of}}\,{\rm{cases}}\,{\rm{that}}\,{\rm{existed}}\,{\rm{in}}\,{\rm{a}}\,{\rm{given}}\,{\rm{age}}\,{\rm{group}}\,}{{\rm{Number}}\,{\rm{of}}\,{\rm{people}}\,{\rm{in}}\,{\rm{the}}\,{\rm{population}}\,{\rm{in}}\,{\rm{a}}\,{\rm{given}}\,{\rm{age}}\,{\rm{group}}\,}$$

For the point estimates Rohlmann confidence intervals were calculated. All data including repeated measurements of SUA in men (n = 306,983) and women (n = 223,738) were used to display SUA concentration according to age.

Multivariate logistic regression models were calculated to estimate the odds ratios (OR) and 95% confidence interval (CI). The statistical software package SAS release 9.4 (SAS Institute, Cary, NC, USA) was used.

### Ethical approval

International, national and state rules were followed implementing the VHM&PP cohort. All participants gave written informed consent for their data to be used for scientific purposes. The study was performed in compliance with the Declaration of Helsinki of the World Medical Association. Full ethical approval for the study was obtained from the Ethics Committee of the State of Vorarlberg.

## Results

**Figure 1:**
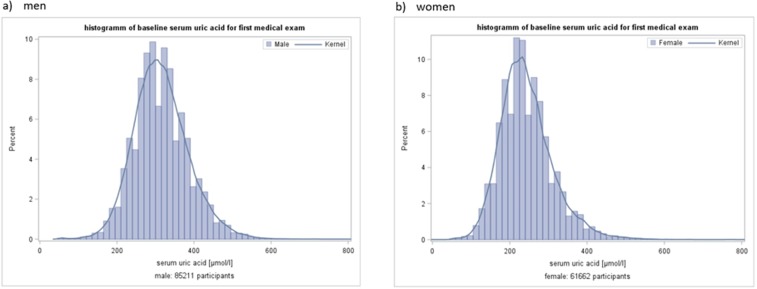
Distribution of serum uric acid concentrations in (**a**) men (n = 85,211) and (**b**) women (n **=** 61,662) at baseline.

The characteristics of the study population are shown in Table 1. At baseline, mean age was 51.5 (SD 13.5) years in women (n = 61,662) and 41.2 (SD 14.6) years in men (n = 85.211). Overall, mean SUA concentration was 284.9 (SD 78.5) µmol/L, with 243.6 (SD 68.3) µmol/L in women and 314.8 µmol/L (SD 71.6) in men. The distribution of SUA concentrations in men and women is depicted in Fig. 1a,b showing a normal Gaussian distribution. A similar distribution was found for the participants aged 50 years and older (Supplementary Fig. 1). Obesity was prevalent in 16.5% of women and in 10.1% of men, hypertension in 47.7% of women and in 40.9% of men. In total, 20.8% of women and 36.6% of men were current smokers.

**Table 1:** Baseline characteristics of the VHM&PP study population 1985–2005.

	mean	SD
**Women (n = 61,662)**		
Age [years]	51.5	13.5
Serum uric acid [μmol/L]	243.6	68.3
BMI [kg/m²]	25.6	4.8
Systolic blood pressure [mmHg]	135.0	23.0
Diastolic blood pressure [mmHg]	82.1	11.4
Blood glucose [mmol/L]	101.4	36.3
Total cholesterol [mmol/L]	228.8	47.5
Triglycerides [mmol/L]	123.3	81.3
Gamma-GT [U/L]	16.7	22.6
	**n**	**%**
Age > 50 years	33861	54.9
Smokers	12848	20.8
Obesity	10196	16.5
Hypertension	29379	47.7
Diabetes	9752	16.0
**Men (n = 85,211)**		
	**mean**	**SD**
Age [years]	41.2	14.6
Serum uric acid [μmol/L]	314.8	71.6
BMI [kg/m²]	25.4	3.6
Systolic blood pressure [mmHg]	132.0	18.8
Diastolic blood pressure [mmHg]	81.6	10.9
Blood glucose [mmol/L]	102.3	36.9
Total cholesterol [mmol/L]	216.5	47.7
Triglycerides [mmol/L]	153.2	122.3
Gamma-GT [U/L]	25.5	36.2
	**n**	**%**
Age > 50 years	22684	26.6
Smokers	31161	36.6
Obesity	8638	10.1
Hypertension	34818	40.9
Diabetes	14610	17.3

### Serum uric acid levels and sex, age and secular trends

**Figure 2:**
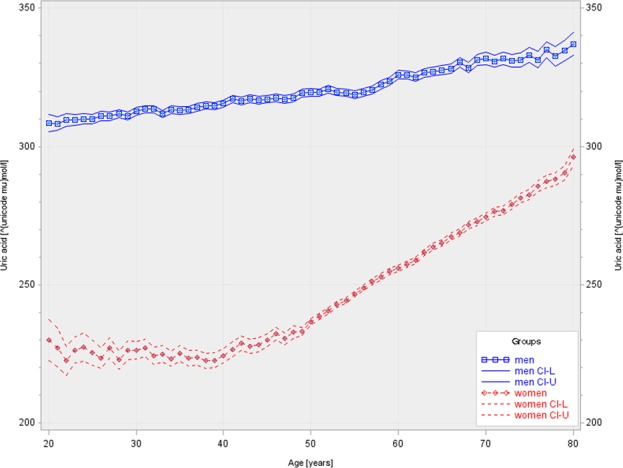
Sex-specific serum uric acid concentration and 95% confidence interval according to age including repeated measurements in men (n **=** 306.983) and women (n **=** 223.738).

Figure 2 shows the mean sex-specific SUA concentrations according to age in 61,662 women and 85,211 men. In women, mean SUA levels stayed fairly constant until the age of 50, namely at approximately 220–230 µmol/L, but started to rise thereafter with a continuous increase of approximately 22 µmol/L per decade to a maximum of about 295 µmol/L at the age of 80. In men, however, SUA increased continuously with age but less strongly. Starting with about 300 µmol/L at age 20, mean SUA levels rose to about 340 µmol/L at age 80, corresponding to an increase of 6.7 µmol/L per decade.

In an additional analysis we determined age-adjusted mean SUA concentrations for women and men in five-year intervals from 1985 to 2005. Mean SUA levels remained virtually constant during these 20 years (Supplementary Fig. 2) indicating that there was no secular trend.

### Prevalence of hyperuricemia

**Figure 3:**
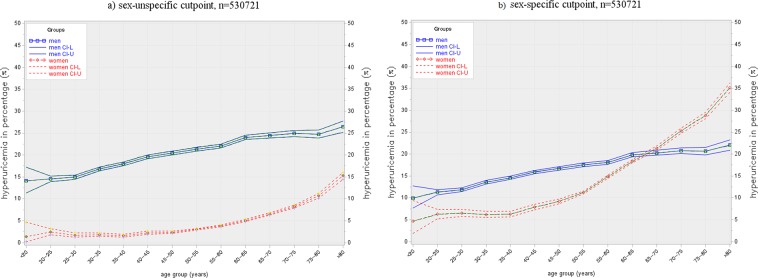
Prevalence of hyperuricemia according to different cut-off points by age and sex, including repeated measurements in men (n = 306.983) and women (n = 223.738). **(a**) cut-off point 360 µmol/L for women and men. **(b**) sex-specific cut-off points, 340 µmol/L for women and 420 µmol/L for men.

At baseline, the age-dependent prevalence of hyperuricemia in women and men according to common and sex-specific cut-off points is shown in Fig. 3a,b. Applying a common cut-off point of 360 µmol/L, the prevalence of hyperuricemia was 4.4% in women and 18.5% in men. According to sex-specific cut-off points, the prevalence of hyperuricemia was higher in women (13.8%) and lower in men (15.1%) compared to the frequencies defined by the common cut-off point. With the common cut-off point of 360 µmol//L for both sexes, the frequency of hyperuricemia increased with age in women and men, but was always higher in men. Contrarily, when using a sex-specific cut-off point for the definition of hyperuricemia (>340 µmol/L in women, >420 µmol/L in men), the prevalence of hyperuricemia increased much more steeply in women after the age of 50, and finally outreached the prevalence in men above the age of about 65.

When repeated measurements during the study period were included for men (n = 306,983) and women (n = 223,738), the prevalence of hyperuricemia increased slightly (common cut-off point: 5.2% in women and 20.4% in men; sex-specific cut-off points: 16.9% in women and 16.7% in men.

### Metabolic risk factors and hyperuricemia

The prevalence of metabolic risk factors according to a common or sex-specific cut-off point of hyperuricemia is shown in Table 2. In men and women with hyperuricemia, smoking, obesity, hypertriglyceridemia and elevated levels of GGT were more prevalent than in participants with normal SUA. The sex-specific correlations between SUA and the metabolic risk factors BMI, BG, TC, TG and GGT are shown in Supplementary Table 1. In a multivariate model adjusted for age (Table 3), the highest odds ratios for hyperuricemia defined by sex-specific cutoffs were observed for obesity (in men odds ratio 3.00, 95% confidence interval 2.85 to 3.16; in women 2.74, 2.60 to 2.88), hypertriglyceridemia (in men 3.12, 3.03 to 3.41; in women 2.85, 2.74 to 2.97) and elevated GGT levels (in men 2.54, 2.38 to 2.75; in women 3.44, 3.24 to 3.65). After additional adjustments for the individual metabolic factors, the above-reported associations were attenuated, but the pattern of statistically significant associations remained similar in men and women.

**Table 2:** Prevalence of metabolic risk factors at different cut-off points for hyperuricemia in the VHM&PP cohort at baseline.

	Men (n **=** 85,211)		Women (n **=** 61,662)	
	Hyperuricemia: SUA ≥ 420 μmol/L		Hyperuricemia: SUA ≥ 340 μmol/L	
	No (n = 72,325)	Yes (n = 12,886)	No (n = 53,184)	Yes (n = 8,478)
**Obesity, n (%)**				
No	66341 (91.8)	10207 (79.2)	45844 (86.2)	5604 (66.1)
Yes	5964 (8.2)	2674 (20.8)	7324 (13.8)	2872 (33.9)
Missing	20 (.)	5 (.)	16 (.)	2 (.)
**Smoking status, n (%)**				
Non-smokers	46301 (64)	7749 (60.1)	41883 (78.8)	6931 (81.8)
Smokers	26024 (36)	5137 (39.9)	11301 (21.2)	1547 (18.2)
**Diabetes mellitus, n (%)**				
No	59661 (83)	10346 (81.1)	45099 (85.4)	6307 (75.5)
Yes	12194 (17)	2416 (18.9)	7708 (14.6)	2044 (24.5)
Missing	470 (.)	124 (.)	377 (.)	127 (.)
**Hypertension, n (%)**				
No	44626 (61.8)	5693 (44.2)	29599 (55.7)	2622 (31)
Yes	27632 (38.2)	7186 (55.8)	23532 (44.3)	5847 (69)
Missing	67 (.)	7 (.)	53 (.)	9 (.)
**Hypertriglyceridemia, n (%)**				
No	59716 (82.6)	7931 (61.7)	48508 (91.2)	6185 (73.1)
Yes	12563 (17.4)	4921 (38.3)	4656 (8.8)	2281 (26.9)
Missing	46 (.)	34 (.)	20 (.)	12 (.)
**Hypercholesterolemia, n (%)**				
No	29240 (40.4)	3298 (25.6)	15446 (29)	1562 (18.4)
Yes	43063 (59.6)	9581 (74.4)	37727 (71)	6909 (81.6)
Missing	22 (.)	7 (.)	11 (.)	7 (.)
**GGT, n (%)**				
Normal	68925 (95.4)	10934 (84.9)	50126 (94.3)	7219 (85.3)
Elevated	3349 (4.6)	1938 (15.1)	3023 (5.7)	1245 (14.7)
Missing	51 (.)	14 (.)	35 (.)	14 (.)

Hyperuricemia was defined as a serum uric level ≥420 μmol/L in men and ≥340 μmol/L in women (sex-specific cut-off point) or as a serum uric level ≥360 μmol/L if not taking gender into account (common cut-off point). Obesity was defined according to the WHO definition with BMI ≥ 30 kg/m². Diabetes mellitus was defined as blood glucose level ≥6.94 mmol/L based on the WHO recommendations. Hypertension was defined as systolic blood pressure ≥140 mmHg or diastolic blood pressure ≥90 mmHg. Hypertriglyceridemia was defined as triglyceride level ≥2.28 mmol/L. Hypercholesterolemia was defined as total cholesterol level ≥5.18 mmol/L. GGT levels ≥61 U/L for men and ≥36 U/L for women defined elevated GGT.

**Table 3:** Factors associated with hyperuricemia (with sex-specific cut-off points).

	Men (n = 85,211)		Women (n = 61,662)	
	OR (95% CI)	OR* (95% CI)	OR (95% CI)	OR* (95% CI)
**Obesity**				
No	Reference	Reference	Reference	Reference
Yes	3.0 (2.85,3.16)	2.0 (1.89,2.11)	2.74 (2.6,2.88)	2.32 (2.2,2.46)
**Smoking status**				
Non-smokers	Reference	Reference	Reference	Reference
Smokers	1.17 (1.1,1.24)	1.06 (1.02,1.11)	1.2 (1.15,1.24)	1.14 (1.06,1.21)
**Diabetes mellitus**				
No	Reference	Reference	Reference	Reference
Yes	1.01 (0.96,1.06)	0.82 (0.78,0.86)	1.34 (1.26,1.42)	1.06 (1,1.13)
**Hypertension**				
No	Reference	Reference	Reference	Reference
Yes	1.92 (1.82,2.02)	1.51 (1.45,1.58)	1.89 (1.82,1.97)	1.43 (1.36,1.52)
**Hypertriglyceridemia**				
No	Reference	Reference	Reference	Reference
Yes	3.21 (3.03,3.41)	2.1 (2.01,2.19)	2.85 (2.74,2.97)	2.4 (2.28,2.53)
**Hypercholesterolemia**				
No	Reference	Reference	Reference	Reference
Yes	1.22 (1.15,1.3)	1.35 (1.29,1.42)	1.81 (1.74,1.9)	0.99 (0.93,1.06)
**GGT**				
Normal	Reference	Reference	Reference	Reference
Elevated	2.56 (2.38,2.75)	2.57 (2.41,2.73)	3.44 (3.24,3.65)	2.04 (1.89,2.2)

OR, logistic analysis was used to calculate odds ratio adjusting for age; OR*, multivariate logistic analysis was used to calculate odds ratio adjusting for age, obesity, smoking status, diabetes, hypertension, hypertriglyceridemia, hypercholesterolemia and elevated GGT. Hyperuricemia was defined using sex-specific cut-off points (SUA level ≥420 μmol/L in men, ≥340 μmol/L in women). Obesity was defined according to the WHO definition with BMI ≥ 30 kg/m². Diabetes mellitus was defined as blood glucose level ≥6.94 mmol/L based on the WHO recommendations. Hypertension was defined as systolic blood pressure ≥140 mmHg or diastolic blood pressure ≥90 mmHg. Hypertriglyceridemia was defined as triglyceride level ≥2.28 mmol/L. Hypercholesterolemia was defined as total cholesterol level ≥5.18 mmol/L. GGT levels ≥61 U/L for men and ≥36 U/L for women defined elevated GGT. CI, confidence interval; OR, odds ratio adjusted for age; OR*, odds ratio multivariate adjusted.

## Discussion

We here report SUA levels in almost 150,000 individuals from the general population with over half a million SUA measurements spanning over two decades, making it to our knowledge the largest study in its field. We found that mean SUA levels are higher in men than in women (314.8 ± 71.6 vs 243.6 ± 68.3 µmol/L) and that they show a normal Gaussian distribution, as already described by others^19^. Furthermore, we found that SUA levels increase with age, but to a different extent in men and women. In men, a modest and linear increase in SUA levels was observed from 20 to 80 years of age. Contrarily, in women SUA levels remained fairly stable until the age of about 50, but continued to increase steeply thereafter. Similar findings were reported in a Western population-based study from Ireland^12^, in two considerably smaller Asian studies from China^20^ and Taiwan^21^, and exclusively for women in the US^22,23^.

The sharp increase in SUA levels in women after the age of 50 might be caused by the hormonal changes experienced during menopause. It is probably due to the loss of the uricosuric effect of estrogens, which have been shown to reduce the protein levels of the urate reabsorptive transporters urate transporter 1 and glucose transporter 9 (Urat1 and Glut9), and of the urate efflux transporter ATP-binding cassette sub-family G member 2 (Abcg2)^24^. Whether the further increase in elderly women is caused by increased SUA production is unknown at present. Next to an increase in body weight, a higher prevalence of impaired kidney function, hypertension, diabetes, hyperlipidemia and use of diuretics might be possible factors leading to higher SUA levels^22^.

Approximately two-thirds of the daily disposal of UA is carried out by the kidneys via glomerular filtration and tubular reabsorption and secretion^2^. With increasing age healthy kidneys are affected by structural changes including global glomerulosclerosis, tubular atrophy and interstitial fibrosis^25^ resulting functionally in a significant decline in glomerular filtration rate with aging in the majority of healthy people^26^. Therefore, a decreased UA clearance with impaired kidney function may contribute to higher SUA levels with increasing age. Whereas there is no relevant sex-specific difference in natural age-related decline of GFR, male patients with chronic kidney disease have a more rapid progress to end-stage kidney disease compared to women^27^. From this, the sex-specific and age-related pattern in SUA levels observed in our study cannot be simply explained by possible differences in kidney function.

### Are SUA levels increasing in the general population?

In the 1920s, mean SUA levels in the US were 202 µmol/L and increased to 372 µmol/L in the 1970s^28^. In Australia, SUA levels increased by 17% in 30- to 40-year-old men from 1959–1980^29^. A very recent study from Ireland found a modest increase of 11 µmol/L from 2006–2014^12^. In the US, SUA levels increased by 9 µmol/L from 1988–1994 to 2007–2008. During the same period the prevalence of hyperuricemia increased by 3.2% and the prevalence of gout by 1.2%^11^. Other studies report an increasing prevalence of hyperuricemia and gout over time. In Italy, over the short period between 2005 and 2009 the prevalence of hyperuricemia increased from 85.4 to 119.3 cases per thousand inhabitants, and the prevalence of gout from 6.7 to 9.1 per thousand^13^. Contrarily, the Nutrition and Health Survey in Taiwan showed a decrease in SUA levels of 10.7 µmol/L in men and 21.4 µmol/L in women from 1993–1996 and from 2005–2008^21^. This was most likely caused by a change in dietary patterns, mainly by reduced consumption of meat and soft drinks. The increase in SUA in most Western countries, to the contrary, is attributed to the obesity epidemic, high intake of meat and seafood, fructose-rich beverages and alcohol^30–33^. In our study population, in contrast to other Western populations, SUA levels remained constant over a period of two decades. The reason for that is unclear at present. Other studies have found that SUA levels and the prevalence of hyperuricemia and gout increase in parallel with BMI, and that adjusting for BMI attenuated the increase over time^11,30^. We found in our cohort a correlation between SUA and BMI. Mean BMI remained fairly constant throughout the study period, which might have partly contributed to the observed stable SUA levels.

It is difficult to compare the prevalence of hyperuricemia between studies due to the differences in the cut-off level used for the definition of hyperuricemia. The most widely used definition is sex-specific with a cut-off point of 420 µmol/L (7.0 mg/dL) in men and 340 µmol/L (5.7 mg/dL) in women^11^. These are statistically derived levels defined as a SUA level of more than two standard deviations above the mean. These levels, however, do not reflect the pathophysiology of UA-induced gout. They also do not consider the age-dependent SUA levels in women. Definitions of hyperuricemia for both sexes range from 360 µmol/L (6.0 mg/dL) to 405 µmol/L (6.8 mg/dL). We and others prefer a unified common cut-off point of 360 µmol/L for several reasons^17,28^. First, sodium-monourate crystal formation occurs in body fluids above a level of 405 µmol/L. A cut-off point of 360 µmol/L would define a safety margin for crystal deposition and the development of gout. Second, the lifetime risk of gout increases above this level^17^. In addition, this cut-off value also corresponds with the target level for treatment of hyperuricemia proposed by the European League against Rheumatism^34^. Using the sex-specific approach, it is clear that the prevalence of hyperuricemia is nearly identical in both men and women, as shown in our study. However, when using a common cut-off value hyperuricemia is seen to be twice as common in men as shown in other studies^13^ or about 4-times more frequent in men as found in our analysis, which comes closer to the male:female gout ratio of about 3 to 4^11,35^.

Our observation that obesity, hypertriglyceridemia and elevated GGT levels were associated with hyperuricemia is consistent with previous findings^36,37^. The mechanisms by which hyperuricemia affects diabetes, cardiovascular and kidney disease are not yet clear. However, there is little evidence for a causal effect. An umbrella review of 136 unique health outcomes revealed only evidence for a clear role of SUA levels for gout and nephrolithiasis^38^.

### Strengths and weaknesses of this study

Despite the very large number of participants and individual SUA measurements making it the largest study in the field, the standard protocol, as well as the long observation period of 20 years, several limitations of our study must be acknowledged. We do not have SUA levels for VHM&PP participants beyond 2005. We therefore do not know whether SUA levels remained constant or have increased since then. Since in women SUA was routinely measured only in those above the age of 50, the results cannot be generalized to younger women. However, the results were robust when we used the repeated measurements. Furthermore, we do not have information on life style factors such as dietary habits or alcohol intake or the concomitant use of medication such as diuretics or allopurinol, which affect SUA levels. In addition, the VHM&PP did not routinely collect data on kidney function, which is another important determinator of SUA levels with lower glomerular filtration rate being associated with higher SUA^39^.

## Conclusion

In a general population of almost 150,000 adults, SUA levels tended to increase in men beyond the age of 20 with an increase of about 6.7 µmol/L per decade. In women, SUA levels remained constant until the age of 50 and then increased by approximately 22 µmol/L per decade. The prevalence of hyperuricemia increased with age. Over 65 years, the prevalence of hyperuricemia defined by sex-specific cut-off points was higher in women than in men. In men and women, hyperuricemia was associated with smoking, obesity, hypertriglyceridemia and elevated levels of GGT. In the clinical setting, a unified common cut-off point for the definition of hyperuricemia for women and men is preferred.

## Supplementary information

Supplementary material.
