# Supplementary material for: Sex- and age-specific variations, temporal trends and metabolic determinants of serum uric acid concentrations in a large population-based Austrian cohort
Source: Sci Rep. 2020 May 5;10:7578. doi: 10.1038/s41598-020-64587-z (PMC7200724; doi:10.1038/s41598-020-64587-z)

**Supplement to:**

**Sex- and age-specific variations, temporal trends and metabolic determinants of serum uric acid concentrations in a large population-based Austrian cohort**

Emanuel Zitt,^1,2,3^ Anton Fischer,^1,3^ Karl Lhotta,^1,2^ Hans Concin,^3^ Gabriele Nagel^3,4^

^1^Vorarlberg Institute for Vascular Investigation and Treatment (VIVIT), Academic Teaching Hospital Feldkirch, Feldkirch, Austria

^2^Department of Internal Medicine III (Nephrology and Dialysis), Academic Teaching Hospital Feldkirch, Feldkirch, Austria

^3^Agency for Preventive and Social Medicine, Bregenz, Austria

^4^Institute for Epidemiology and Medical Biometry, Ulm University, Ulm, Germany

**Supplementary Table 1:**

Pearson correlations between metabolic factors at baseline

**Supplementary Figure 1:** Distribution of the serum uric acid concentration in participants aged 50 years and older (men n=33,861 and women n= 22,684)

**Supplementary Figure 2:**

Sex-specific mean serum uric acid concentration and 95% confidence interval according to time period, adjusted for age including repeated measurements in men (n= 306.983) and women (n= 223.738)

**Supplementary Table 1:** Pearson correlations between metabolic factors in a) women (N=61,662) and b) men (N=85,211) at baseline

1. **women**

| **Pearson correlation coefficients**  **prob > \|r\| under H0: Rho = 0**  **Number of Observations** | | | | | | | |
| --- | --- | --- | --- | --- | --- | --- | --- |
|  | **Age baseline [years]** | **BMI [kg/m²]** | **Blood glucose [mmol/L]** | **Total cholesterol [mmol/L]** | **Triglycerides [mmol/L]** | **Gamma-GT [U/L]** | **Serum uric acid [µmol/L]** |
| **Age baseline [years]** | 1.00000  61662 | 0.21288 <.0001 61644 | 0.29959 <.0001 61158 | 0.39494 <.0001 61644 | 0.21115 <.0001 61630 | 0.10530 <.0001 61615 | 0.22761 <.0001 61662 |
|  |  |  |  |  |  |  |  |
| **BMI  [kg/m²]** |  | 1.00000  61644 | 0.19593 <.0001 61140 | 0.12655 <.0001 61626 | 0.26818 <.0001 61612 | 0.11177 <.0001 61597 | 0.30709 <.0001 61644 |
|  |  |  |  |  |  |  |  |
| **Blood glucose [mmol/]** |  |  | 1.00000  61158 | 0.18689 <.0001 61142 | 0.22861 <.0001 61127 | 0.11649 <.0001 61113 | 0.11484 <.0001 61158 |
|  |  |  |  |  |  |  |  |
| **Total cholesterol [mmol/L]** |  |  |  | 1.00000  61644 | 0.35846 <.0001 61621 | 0.10746 <.0001 61605 | 0.16589 <.0001 61644 |
|  |  |  |  |  |  |  |  |
| **Triglycerides [mmol/L]** |  |  |  |  | 1.00000  61630 | 0.19213 <.0001 61593 | 0.27557 <.0001 61630 |
|  |  |  |  |  |  |  |  |
| **Gamma-GT [U/L]** |  |  |  |  |  | 1.00000  61615 | 0.16407 <.0001 61615 |
|  |  |  |  |  |  |  |  |
| **Serum uric acid [µmol/L]** |  |  |  |  |  |  | 1.00000  61662 |

1. **men**

| **Pearson correlation coefficients**  **prob > \|r\| under H0: Rho = 0**  **Number of Observations** | | | | | | | |
| --- | --- | --- | --- | --- | --- | --- | --- |
|  | **Age baseline [years]** | **BMI [kg/m²]** | **Blood glucose [mmol/L]** | **Total cholesterol [mmol/L]** | **Triglycerides [mmol/L]** | **Gamma-GT [U/L]** | **Serum uric acid [µmol/L]** |
| **Age baseline [years]** | 1.00000  85211 | 0.24008 <.0001 85186 | 0.25769 <.0001 84617 | 0.31978 <.0001 85182 | 0.10967 <.0001 85131 | 0.11948 <.0001 85146 | 0.06207 <.0001 85211 |
|  |  |  |  |  |  |  |  |
| **BMI  [kg/m²]** |  | 1.00000  85186 | 0.16277 <.0001 84592 | 0.23663 <.0001 85157 | 0.27895 <.0001 85106 | 0.14610 <.0001 85121 | 0.25213 <.0001 85186 |
|  |  |  |  |  |  |  |  |
| **Blood glucose [mmol/]** |  |  | 1.00000  84617 | 0.17899 <.0001 84589 | 0.16037 <.0001 84545 | 0.12117 <.0001 84555 | 0.00228 0.5073 84617 |
|  |  |  |  |  |  |  |  |
| **Total cholesterol [mmol/L]** |  |  |  | 1.00000  85182 | 0.39374 <.0001 85116 | 0.18815 <.0001 85126 | 0.18917 <.0001 85182 |
|  |  |  |  |  |  |  |  |
| **Triglycerides [mmol/L]** |  |  |  |  | 1.00000  85131 | 0.25537 <.0001 85086 | 0.23553 <.0001 85131 |
|  |  |  |  |  |  |  |  |
| **Gamma-GT [U/L]** |  |  |  |  |  | 1.00000  85146 | 0.18458 <.0001 85146 |
|  |  |  |  |  |  |  |  |
| **Serum uric acid [µmol/L]** |  |  |  |  |  |  | 1.00000  85211 |

**Supplementary Figure 1:** Distribution of the serum uric acid concentration in participants aged 50 years and older (men n=33,861 and women n= 22,684)


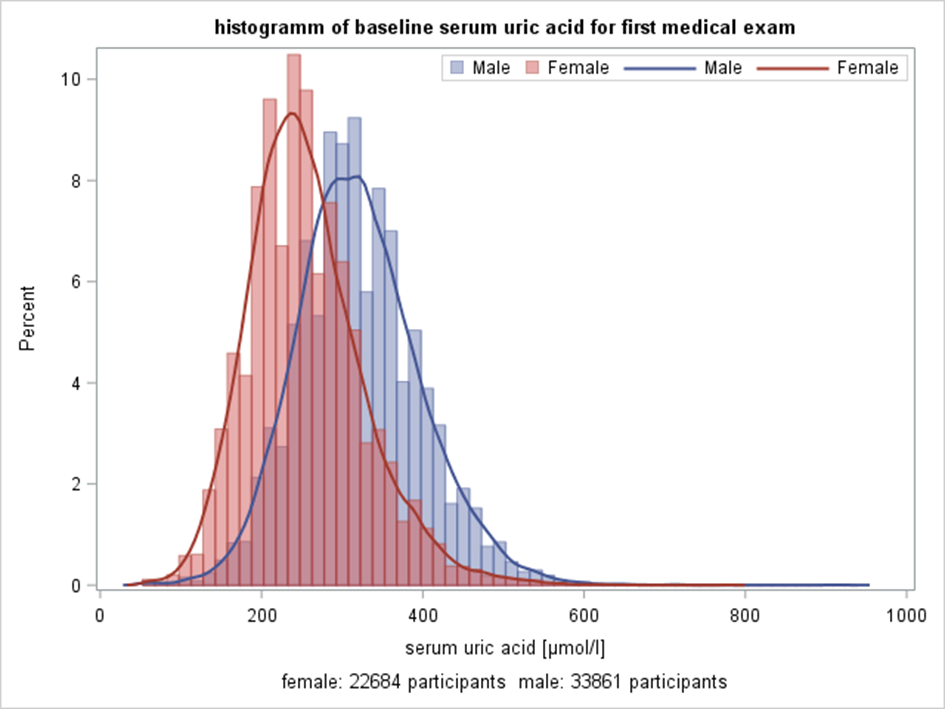


**Supplementary Figure 2:** Sex-specific mean serum uric acid concentration and 95% confidence interval according to time period, adjusted for age including repeated measurements in men (n= 306.983) and women (n= 223.738)


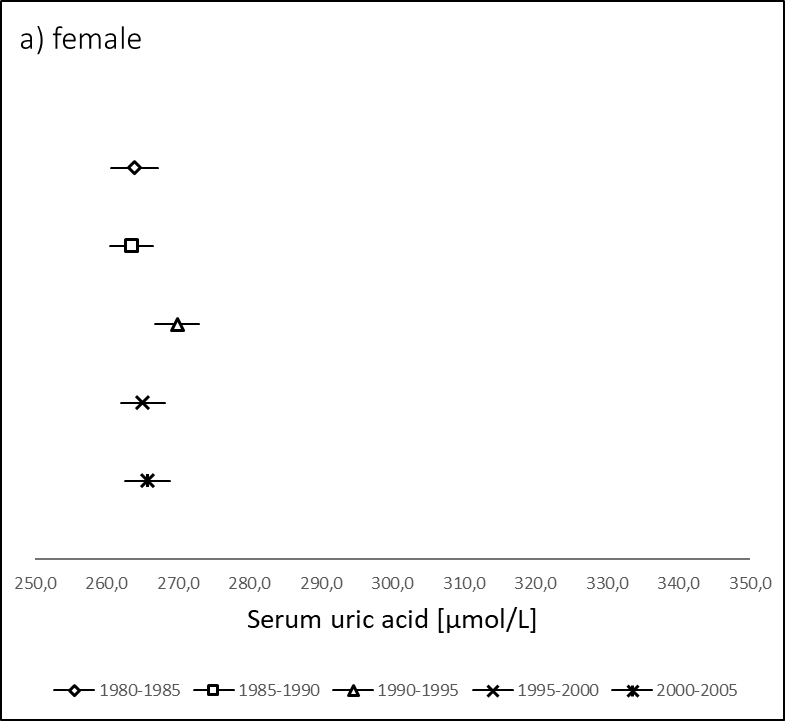

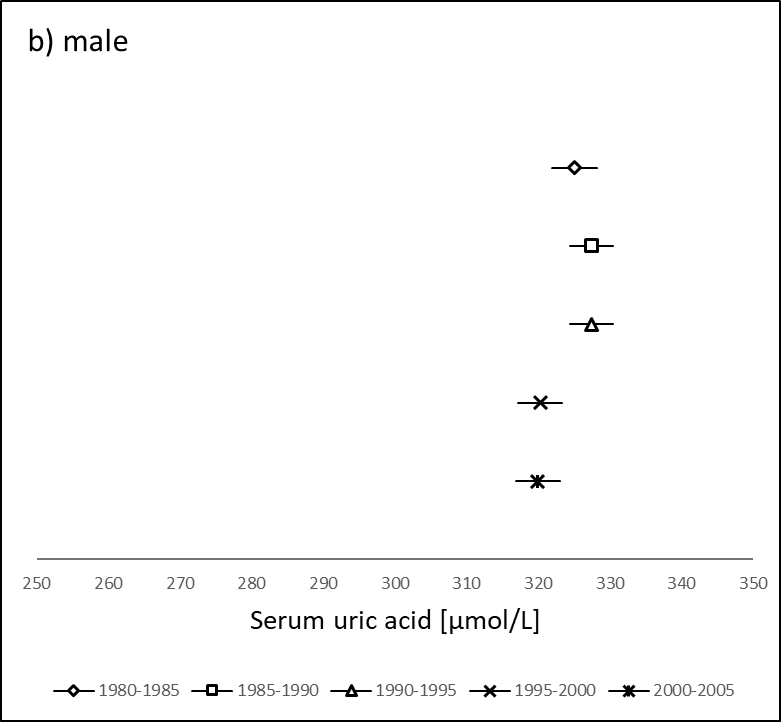

Supplement: Supplementary file 1 — Supplementary material. [file 41598_2020_64587_MOESM1_ESM.docx]
